# Plasticity in oviposition and foraging behavior in the invasive pest *Drosophila suzukii* across natural and agricultural landscapes

**DOI:** 10.1002/ece3.9713

**Published:** 2023-01-06

**Authors:** Johanna E. Elsensohn, Hannah J. Burrack

**Affiliations:** ^1^ Department of Entomology and Plant Pathology North Carolina State University Raleigh North Carolina USA; ^2^ USDA‐ARS Appalachian Fruit Research Station Kearneysville West Virginia USA; ^3^ Department of Entomology Michigan State University East Lansing Michigan USA

**Keywords:** crop domestication, forest, non‐native species

## Abstract

The effects and extent of the impacts of agricultural insect pests in and around cropping systems is a rich field of study. However, little research exists on the presence and consequence of pest insects in undisturbed landscapes distant from crop hosts. Research in such areas may yield novel or key insights on pest behavior or ecology that is not evident from agroecosystem‐based studies. Using the invasive fruit pest *Drosophila suzukii* (Matsumura) as a case study, we investigated the presence and resource use patterns of this agricultural pest in wild blackberries growing within the southern Appalachian Mountain range of North Carolina over 2 years. We found *D. suzukii* throughout the sampled range with higher levels of infestation (*D. suzukii* eggs/g fruit) in all ripeness stages in natural areas when compared with cultivated blackberry samples, but especially in under‐ripe fruit. We also explored a direct comparison of oviposition preference between wild and cultivated fruit and found higher oviposition in wild berries when equal weights of fruit were offered, but oviposition was higher in cultivated berries when fruit number was equal. Forest populations laid more eggs in unripe wild‐grown blackberries throughout the year than populations infesting cultivated berries. This suggests *D. suzukii* may change its oviposition and foraging behavior in relation to fruit type. Additionally, as *D. suzukii* exploits a common forest fruit prior to ripeness, further research is needed to explore how this affects wild food web dynamics and spillover to regional agroecosystems.

## Graphical abstract

The invasive *Drosophila suzukii* infests both wild‐growing and cultivated blackberry fruit. The infestation pattern differs between the two types, where wild‐growing berries received more eggs by weight and in more underripe stages while cultivated fruit received more eggs per berry for the two ripest stages. These findings suggest oviposition behavior may differ by fruit type and environment.

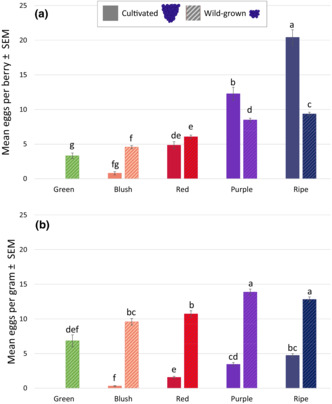

## 1 INTRODUCTION

The niche breadth of polyphagous insect pests can be expansive due to a number of biological and abiotic factors including the ability to exploit diverse host types in heterogenous environments and the capacity to respond to changing conditions over time (Kennedy & Storer, 2000; Little et al., 2020; Sakai et al., 2001). Species with broad host ranges also tend to have an outsized impact on crops when compared with monophagous or oligophagous insect species (Ward & Spalding, 1993). It is reasonable to assume these substantial impacts may also occur in non‐crop areas. Considerable research has been conducted in semi‐natural lands adjacent to or near affected crops, as these are the areas thought to be highly influential to agroecosystem dynamics (Kennedy & Storer, 2000; Mazzi & Dorn, 2012; Rand et al., 2006).

For polyphagous pests, non‐crop host plants occur throughout the landscape, including places far removed from agriculture. These areas, such as forests, are rarely assessed for the presence of agricultural insect pests unless they are also considered a forest pest, such as pear thrips, *Taeniothrips inconsequens* (Uzel) (Teulon et al., 1998), or the spotted lanternfly, *Lycorma delicatula* White (Barringer & Ciafré, 2020). Nevertheless, studying non‐forest crop pests in remote areas might be important for a number of reasons. First, these insects may impact forest food web dynamics through resource use competition of common host plants. Second, if these remote locations host established populations of agricultural pests, then they may be a source for seasonal migrants into cultivated areas. Third, understanding pest behavior outside of the agroecosystem may yield novel insights into pest behavior and ecology that may not be evident in highly human‐influenced agricultural areas. Finally, such insights can then be used to improve modeling predictions for current and future range expansions.

Distribution modeling is a common way to model invasive species and is based on known life history traits and occurrence. However, ground truthing to inform or verify model‐based inferences (e.g., likelihood of occurrence or density) often fails to venture outside of areas where the pest is causing direct economic damage, namely cropland in this case. Failure to fully verify these distribution models limits their usefulness and insight (Fitzpatrick et al., 2007; Sarquis et al., 2018; Wright et al., 2006). Furthermore, niche divergence may occur more readily in areas with more diverse habitat and can be indicative of ecological changes such as invasive species establishment, food web disruption, or climate change (Wright et al., 2006).

*Drosophila suzukii* (Matsumura) is a highly cosmopolitan agricultural pest of berry crops. Native to East Asia, *D. suzukii* was limited in spread until 2008 when accidental introductions led to a range expansion into Europe and continental North America, and in subsequent years to South America, Western Asia and most recently in Africa (Calabria et al., 2012; Deprá et al., 2014; Hassani et al., 2020; Hauser, 2011; Parchami‐Araghi et al., 2015). Ripe fruit from cultivated berry crops and wild‐growing native and non‐native plant species serve as oviposition sites for female *D. suzukii* and nutritional resources for all life stages. The presence and movement of this fly has been well‐studied in croplands and nearby disturbed or wooded areas that serve as potential refuge sites and often contain susceptible hosts (Bellamy et al., 2013; Elsensohn & Loeb, 2018; Klick et al., 2016; Lee et al., 2015; Santoiemma et al., 2018). Some host plant species are regionally common and can be found well outside agroecosystems, including in backyards, roadsides, woods, and fields (e.g., Ballman & Drummond, 2017; Mitsui et al., 2010; Poyet et al., 2014).

Several ecological models were created to assess the current and future distribution of *D. suzukii* around the world (de la Vega & Corley, 2019; dos Santos et al., 2017; Fraimout & Monnet, 2018; Gutierrez et al., 2016; Ørsted & Ørsted, 2019). One species distribution model using global occurrence data indicated a higher likelihood of occurrence in the southern Appalachian Mountains of the eastern United States than in surrounding areas (Ørsted & Ørsted, 2019). Contrastingly, a physiologically‐based demographic model estimated a lower *D. suzukii* density in the same area (Gutierrez et al., 2016). Much of this region of the Appalachian Mountains, which ranges in elevation from 900 to 1850 m, is designated as federally protected National Forest land. No commercial plantings of cultivated *D. suzukii* hosts are known to occur within this area, although *D. suzukii*‐susceptible *Vaccinium* and *Rubus* spp. native to North America grow well here (Powell & Seaman, 1990).

To date, no studies have sought to ground truth model‐predicted occurrence sites sparsely populated by humans as potential population sources of *D. suzukii*. In Europe, altitudinal studies demonstrated an established presence of *D. suzukii* in high elevation, mountainous locations (Santoiemma et al., 2019; Tait et al., 2018), and documented recapturing marked adults over 9 km from the release site (Tait et al., 2018). This distance is suggestive of weather‐assisted movement, as flight mill tests show the flight capacity of adults is <2 km (Wong et al., 2018). Insect dispersal through wind patterns is documented in several pest species (Compton, 2002; Hoelscher, 1967; Moser et al., 2009) and has been postulated as a potential means of yearly recolonization by *D. suzukii* at northern U.S. latitudes after winter temperatures kill the vast majority of overwintering flies (Panel et al., 2018; Rossi‐Stacconi et al., 2016; Wallingford et al., 2018). Localized *D. suzukii* movement from shrubby or wooded landscapes into crop fields is well documented (Klick et al., 2016; Pelton et al., 2016; Tonina et al., 2018). Uncultivated and cultivated areas can be exploited concurrently or consecutively throughout the year, especially in areas where adults are caught year‐round (Ballman & Drummond, 2017; Elsensohn & Loeb, 2018; Santoiemma et al., 2018). Uncultivated areas can enlarge or sustain pest populations that could spill back into crop areas through short or long‐distance movement, and vice versa.

Some non‐crop hosts may be preferred oviposition sites for female *D. suzukii* or offer better nutritional resources needed for larval development. Comparative work exploring oviposition preference between crop and non‐crop host species found that preference depended on the specific fruit combinations used (Diepenbrock et al., 2016; Lee et al., 2015). The first direct comparison of *D. suzukii* oviposition preference between wild and cultivated fruit of the same crop type found that females laid more eggs into cultivated than wild blueberries (Rodriguez‐Saona et al., 2019). However, these results may be confounded by differences in fruit size, weight, and surface area between domesticated and wild relatives.

Laboratory research into oviposition preference as a factor of fruit ripeness stage revealed that the ripe stage was the most preferred for oviposition while progressively under‐ripe stages received fewer or no eggs (Kamiyama & Guédot, 2019; Lee et al., 2011). In a field setting, fewer adults emerged from blackberry fruit infested during under‐ripe stages than fruit infested later at the ripe stage (Swoboda‐Bhattarai & Burrack, 2015). These results align with laboratory studies that show a survival hierarchy with ripe fruit producing the lowest mortality rate (Bernardi et al., 2017; Kamiyama & Guédot, 2019; Lee et al., 2011).

To better understand *D. suzukii* oviposition preference and general resource use in areas unaffected by spillover dynamics, we conducted an elevational gradient study in the southern Appalachian Mountain region. Here, modeling predictions are uncertain, but non‐crop hosts are common. Over 2 years, we visited three natural areas and one roadside tract surrounded by National Forest lands in western North Carolina to collect wild‐growing fruit at different stages of ripeness. The main objectives of this study were to: (1) document the presence or absence of *D. suzukii* in the unpopulated areas; (2) compare seasonal host use patterns of wild and cultivated blackberry fruits; and (3) assess host preference between wild and crop fruit in a laboratory setting.

## 2 METHODS

### 2.1 2017 Field collections

We visited four locations in North Carolina where large wild blackberry stands (>15 canes/10 m) were established: Southern Nantahala Forest in Macon County; Cherokee National Forest in Avery County, and Joyce Kilmer–Slickrock Wilderness Area (JKWA) and along the Cherohala Skyway in Graham County (Table S1). Required permits to sample in these places were obtained from the appropriate agencies. We simultaneously sampled cultivated blackberries var. Ouachita from two research stations that were located in the mountainous regions of western NC. The cultivated blackberry plots received fungicides as needed, but no insecticides or acaricides were applied for the duration of the study. Wild sampling sites were determined by location and density of blackberry plants and separated by a distance of at least 1 km. Fruit collection began when wild‐growing fruit appeared almost full size (subjectively determined by drupelet size) but were still green. Sites were resampled every 2–3 weeks until no ripe fruit were available.

At each site, blackberry plants within a radius of 10 m were sampled for fruit at the following ripeness stages: green, blush (reddish green), red, purple, and ripe. Two research station plantings of cultivated blackberries were sampled during the same week as wild collections, but only ripe fruit were collected at research farms after the first visit due to low fruit set that year. Up to 20 fruits of each stage were sampled at each site as available, grouped in breathable bags, and transported to the lab in a cooler (4°C). Fruits were collectively weighed by sample group and examined under a dissecting microscope for the number of *D. suzukii* eggs laid per berry. *Drosophila suzukii* eggs were distinguished from other potential fruit‐infesting flies by counting the number of respiratory filaments per oviposition site (Hauser, 2011). Although *Drosophila melanogaster* and *D. simulans* eggs also possess only two filaments per egg, we collected fruits before they were susceptible to oviposition by these two species. As opposed to *D. suzukii*, both *D. melanogaster* and *D. simulans* are saprophytic and lack the sharp and sclerotized needed to deposit eggs into ripening fruit (Atallah et al., 2014). Other plant species growing adjacent to blackberry plants with ripe fruit that appeared susceptible to *D. suzukii* were collected at random and similarly checked for infestation. All plant species were identified using Weakley (2006).

### 2.2 2018 Field collections

JKWA was chosen as the focal wild fruit collection location in 2018. Wild blackberries were collected along a trail approximately every 50 m change in elevation at five elevations along a single trail (1430–1630 m) with up to 20 fruits per ripeness stage collected per elevation (Table S1). We could only utilize one research station planting in 2018 because the other planting was removed at the end of the 2017 growing season. However, all ripeness stages were assessed for infestation at this location, as available. All other sampling methods remained the same as in the previous year.

### 2.3 Oviposition preference

Exclusion netting (a mesh bag approximately 100 × 150 mm) was placed around single infructescences after petal fall in both years on wild and cultivated blackberry bushes. Netting bags were secured at the base of the cluster with a foam strip encircled by a plastic zip tie to ensure a tight seal to prevent insect entry but not damage the plant. Fruit were monitored, and when ripe fruit were observed in both cultivation types, all netted, ripe berries were collected at a single wooded and cultivated site on the same day and brought back to the lab. The following day, fruit were examined under a microscope to verify a lack of insect or mechanical damage. A two‐choice bioassay was set up using equal weights of cultivated and wild blackberries placed in 35 × 10 mm petri dishes in the bottom of a 473‐ml plastic container. Two 5–7‐day old females from the laboratory colony (see Hardin et al., 2015) were added to the container and removed after 90 min and the number of eggs per berry was counted. A separate two‐choice assay compared oviposition preference between a single cultivated and single wild blackberry fruit using the same protocol.

### 2.4 Statistical analysis

Data were analyzed using SAS v. 9.4 (SAS Institute, Cary, NC). Samples that comprised fewer than 10 fruits were excluded from the analysis. We define the following variables used in the analysis: ‘week’ represents time after the first sample was collected and held constant for both years; ‘cultivation type’ denotes whether the berry was wild‐grown or cultivated; ‘eggs per berry’ is the mean number of eggs per fruit while ‘eggs per gram’ was calculated using the number of eggs per berry divided by the average per berry weight of each sample group. The ‘eggs per gram’ value was log transformed to adjust for assumptions of normality.

Unless noted otherwise, we used a generalized mixed model (GLIMMIX) with a log normal distribution. Adjusted means were compared using the Tukey–Kramer adjustment. To examine infestation, eggs per berry or eggs per gram was used as the dependent variable, ripeness stage and cultivation type were considered fixed effects, with year, elevation nested within year, location, and week nested within year as random effects. For weekly infestation rates, eggs per gram was used as the dependent variable, week, ripeness stage and cultivation type were considered fixed effects, and year, location, and berry nested in location by week were random effects. The proportion of infested fruit at each sampling point (ripeness stage/location/week) was calculated as the number of berries with one or more eggs divided by the total number of berries in that sample group.

The effect of elevation was assessed for JKWA samples from 2018 with elevation, ripeness stage and week as fixed effects. Data were fitted to a normal distribution via Proc GLIMMIX with cultivation type, ripeness stage, and their interaction as fixed effects and location, year, and elevation nested within location as random effects. The interaction between ripeness stage and elevation was not significant, so comparing between elevations was not illuminating. Instead, we ran separate models for each elevation to simplify the mean separation values within each elevation.

Oviposition preference was calculated as the proportion of eggs laid in either the wild or cultivated berries divided by the total number of eggs laid in each replicate. These proportion data were then evaluated with a paired Student's *t*‐test; replicates with non‐responding flies (those which did not lay eggs during the experimental period) were removed from analysis.

## 3 RESULTS

### 3.1 Field infestation

**FIGURE 1:**
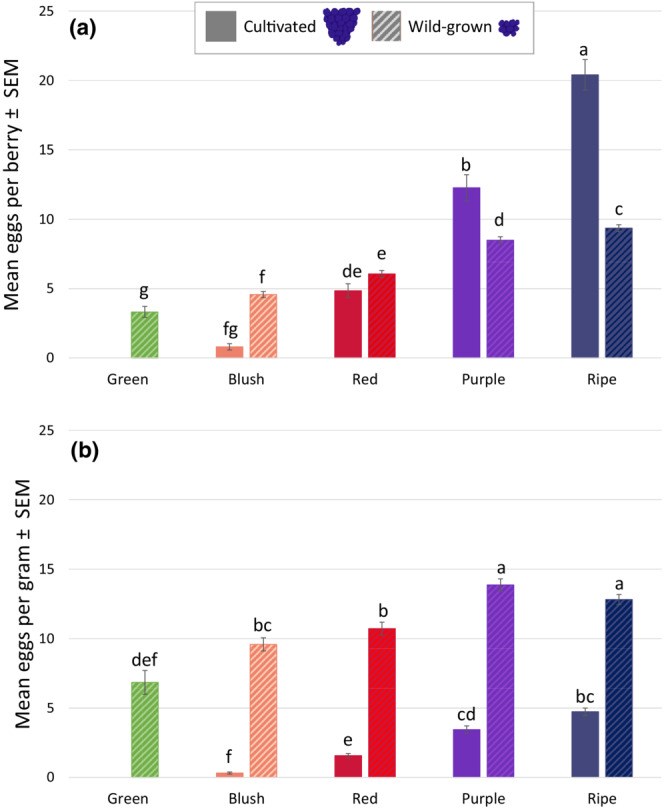
(a) Mean ± SEM number of *Drosophila suzukii* eggs/berry averaged across all collection locations and dates. Cultivated purple and ripe fruit contain more eggs than the wild type. (b) Mean ± SEM eggs/g of fruit show higher infestation levels in wild fruit. Raw means are presented, with adjusted means used for mean separation. Mean values within each pane indicated by the same letter are not significantly different from each other (alpha = 0.05).

Both ripeness stage and cultivation type had a significant effect on the number of eggs per berry (Figure 1a; ripeness stage × cultivation type: *p* < .0001, *F*_3,1951_ = 69.72, GLIMMIX). In both years, cultivated ripe and purple berries carried more eggs than wild berries at the same ripeness stage, while infestation in red, blush and green berries were more similar. However, cultivated berries on average were 3–4‐fold the weight of wild ones, and after accounting for weight, wild berries contained more eggs per gram than cultivated berries at all ripeness stages (Figure 1b; ripeness stage × cultivation type: *p* < .0001, *F*_3,1786_ = 9.08).

**FIGURE 2:**
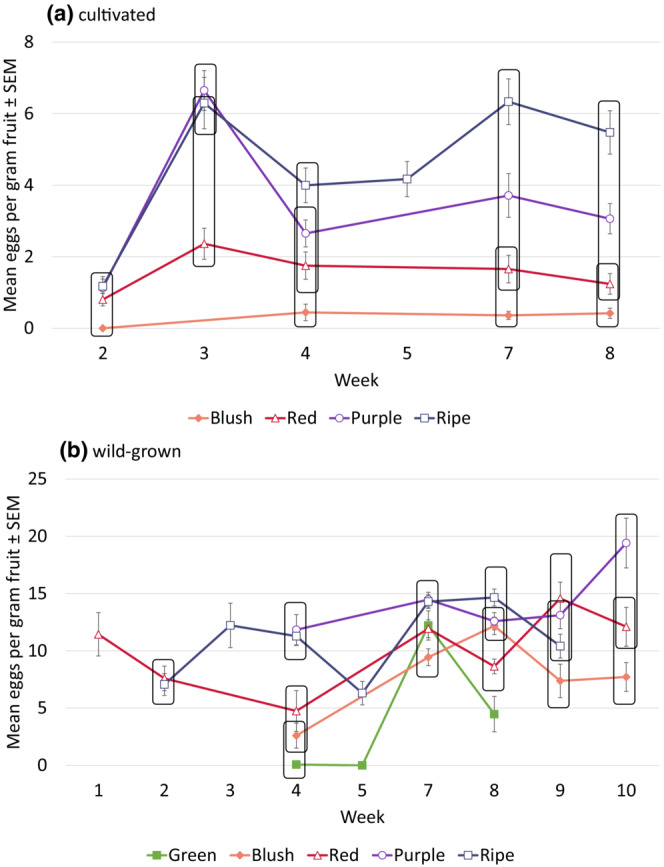
Weekly infestation rates ± SEM across both years for (a) cultivated berries and (b) wild berries. Symbols denote sample points; not all ripeness stages were available to be collected each week. Raw means are presented, with adjusted means used for mean separation. Mean values within the same quadrangle are not significantly different from each other (alpha = 0.05).

There was a three‐way interaction effect of ripeness stage, week, and cultivation type on infestation per gram of fruit over time (ripeness stage × week × cultivation type: *p* = .01, *F*_7,1759_ = 2.52, GLIMMIX). Cultivated berries appeared to maintain a consistent infestation pattern throughout the season, with ripe fruit containing the most eggs and the blush stage (least ripe stage collected) having the fewest eggs (Figure 2a). In contrast, the correlation between infestation and ripeness stage in wild fruit is much less clear (Figure 2b), even though ripeness overall was a significant factor (ripeness stage: *p* < .0001, *F*_4,1759_ = 91.81). We sampled two habitat types (woods and roadside) for wild‐growing berries, and unexpectedly the average infestation between the two types were similar (Table S2).

**FIGURE 3:**
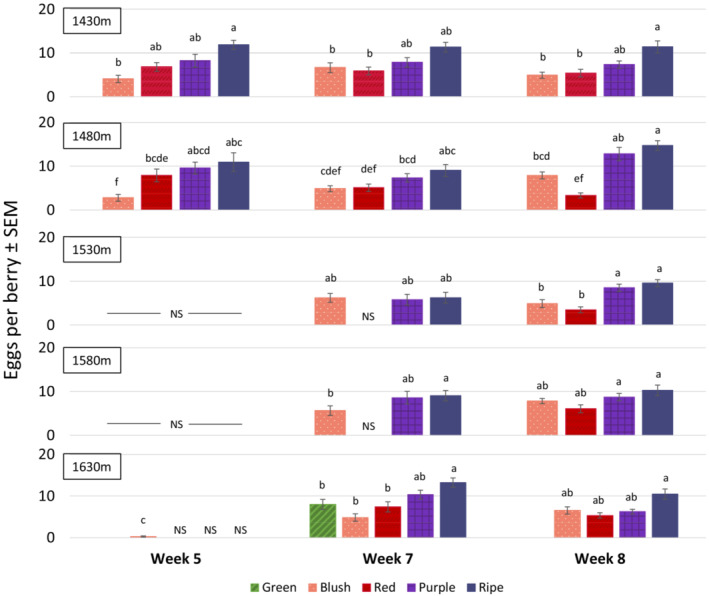
Weekly infestation averages in the 2018 elevational transects. Mean values within the same elevation indicated by the same letter are not significantly different from each other, alpha = 0.05. NS = zero or fewer than 10 berries were sampled at that collection point.

Focusing only on the 2018 wild fruit samples collected at different elevations, effects from elevation and ripeness stage were each significant (elevation: *p* < .0001, *F*_4,851_ = 6.21; ripeness stage: *p* < .0001, *F*_4,851_ = 40.48, GLIMMIX), but their interaction was not, suggesting that all elevations were infested to a similar degree (elevation × ripeness stage: *p* = .35, *F*_12,851_ = 1.11). There is evidence for differential timing of infestation, as the three‐way interaction among all variables was significant (Figure 3; elevation × ripeness stage × week: *p* = .002, *F*_13,851_ = 2.49). Lower elevations had available fruit for collection 2 weeks before higher elevations. In general, the blush and ripe stage differed in infestation at the various elevations and over time. At several timepoints (Week 7: 1430 m, 1480 m 1530 m) and in Week 8 (1430 m, 1480 m, 1630 m), the red stage also had significantly fewer eggs than the ripe stage.

To examine differences in the pattern of infestation among sample types, we calculated the percentage of berries that contained at least one egg per sample group. Ripe and purple wild berries and ripe cultivated berries had above a 95% mean infestation across all timepoints (Table 1). Cultivated and wild fruits of the same ripeness stage (cultivation type: *p* = .0625, *F*_1, 55_ = 3.61) were not significantly different from each other, although green wild and blush fruits of both types were significantly less infested overall than those at the ripe stage (cultivation type × ripeness stage: *p* < .0001, *F*_3,55_ = 8.62). A weekly breakdown shows near 100% infestation in wild ripe and purple fruits throughout the sampling period, but more variable infestation in fruit at earlier ripeness stages (Figure S1). Sampling other wild‐growing fruits found near wild blackberry canes revealed a range of infestation patterns (Table S3). Plant species phylogenetically close to known *D. suzukii* host plants were more likely to be infested than those more distant phylogenetically.

**TABLE 1:** Average percentage of berries per sample that were infested with at least one egg for each cultivation and ripeness type.

Growth type	Ripeness stage	No. collection points	Total *N*	Mean percentage infestation ± SE
Wild	Green	8	119	55.7 ± 16.0 ab
Wild	Blush	19	350	81.0 ± 4.6 bc
Wild	Red	20	378	93.6 ± 2.8 bd
Wild	Purple	29	496	96.4 ± 2.5 bd
Wild	Ripe	30	562	98.6 ± 0.7 d
Cultivated	Blush	4	80	28.8 ± 10 a
Cultivated	Red	5	100	82.0 ± 4.1 bd
Cultivated	Purple	5	101	94.0 ± 4.8 bd
Cultivated	Ripe	10	185	98.0 ± 1.5 cd

*Note*: Mean separations were determined by ANOVA followed by Tukey Kramer adjustment. Mean values followed by the same letter are not significantly different from each other, alpha = 0.05.

### 3.2 Oviposition preference

**FIGURE 4:**
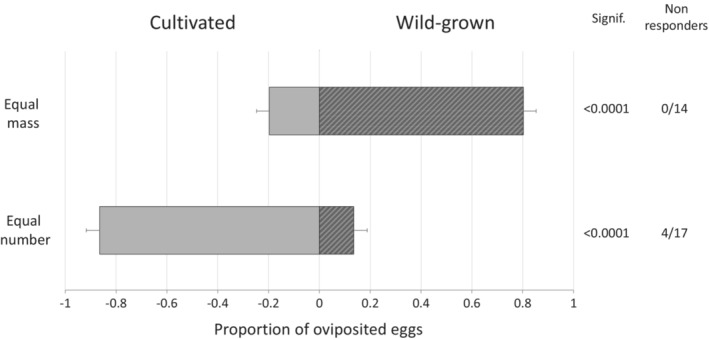
Mean proportion of eggs ± SEM laid into cultivated or wild‐grown blackberries. Females show opposite oviposition preferences when given an equal mass or equal number of cultivated and wild fruit. Student's *t*‐test: Equal mass *t*_13_ = 5.72; equal number *t*_12_ = −4.65, alpha = 0.05.

When exposed to equal masses of cultivated and wild blackberries, laboratory‐reared female *D. suzukii* laid more eggs in wild fruit (Figure 4; 0.80 ± 0.05 SEM for wild, 0.20 ± 0.05 for cultivated; *p* < .0001, *t*_13_ = 5.72, Student's *t*‐test). The number of wild berries per replicate varied (1–9 berries/replicate, median = 5 berries) due to the variability in the weights of the cultivated berries (0.82–4.41 g/berry, median weight = 3.38 g). However, when exposed to a single berry of each type, that preference was reversed (Figure 4; 0.14 ± 0.05 for wild, 0.86 ± 0.05 for cultivated, *p* < .0001, *t*_12_ = 4.65).

## 4 DISCUSSION

By examining the behavior of an agricultural pest in a remote, non‐crop setting, we can gain a better understanding of the ecological, behavioral, and physiological plasticity of the insect. First, the high infestation rates (eggs/g fruit) observed in the forested locations suggest these areas are highly suitable to *D. suzukii* establishment. Distribution models for agricultural pests are trained on occurrence data at a regional or global scale, however oftentimes, the available data are collected in a non‐random manner. For instance, *D. suzukii* sampling in the United States has mostly occurred in and around susceptible cropping areas. As demonstrated with these data, a common criticism of presence‐only models is that they do not adequately extrapolate to novel areas (Elith & Leathwick, 2009; Roach et al., 2017). Models trained on *D. suzukii* occurrence data from North and South America performed worse in this ground truthing exercise than the model trained on a global data set, suggesting improvements could result from more diverse sampling schemes.

Second, wild blackberries were as or more susceptible to *D. suzukii* oviposition at all ripeness stages than the cultivated blackberries in this study. In an evolutionary sense, cultivated crops are thought to be more exploitable by insect pests than wild relatives due to human‐mediated plant domestication selecting against plant defensive traits (Chen et al., 2015; Whitehead et al., 2017). For instance, bitter‐tasting secondary metabolites that deter insect feeding are greatly reduced in domesticated plant species (Wink, 1988). Generally speaking, domesticated fruits are also much larger than their wild ancestors, and frugivores tend to prefer larger fruits, lending support to this plant domestication‐reduced defense hypothesis. Indeed, female *D. suzukii* laid more eggs into cultivated blueberries than wild ones (Rodriguez‐Saona et al., 2019). While we also saw the greatest eggs per berry in cultivated ripe and purple fruit, there were significantly more eggs in wild berries after mass was taken into account. Although we do not know how larval competition affects survivability to adulthood in these natural areas, laboratory studies have shown high *D. suzukii* larval densities can lower mean survivorship, however host quality mediates this effect. For example, resource competition is more pronounced when larvae are exposed to either low protein or low carbohydrate diets (Hardin et al., 2015), survivorship was highest when protein: carbohydrate diet ratios mirrored *D. suzukii* oviposition hosts (Young et al., 2018), and larvae developing in high pH blueberries (relative to others tested) experienced a greater proportion of emerging adults and shorter development times (Molina et al., 2020). Additionally, the availability of yeast species to developing larvae affect offspring performance and often influences female oviposition behavior (Bellutti et al., 2018; Hamby & Becher, 2016; Young et al., 2018).

In our case, both genotype and environmental factors impact the quality and quantity of common *Rubus* attributes, such as soluble solid and phytochemical content (Anttonen & Karjalainen, 2005; Van de Velde et al., 2016). In a comparative study, wild blackberries contained significantly higher pH levels, total soluble solids, and total phenolic compounds than the cultivated varieties tested (Yilmaz et al., 2009). Non‐comparative studies that tested different varieties of wild and cultivated blackberries support those results (Cosmulescu et al., 2017; Van de Velde et al., 2016). Taken together, the higher number of eggs per gram fruit in the wild berries may be reflective of an environment better suited for *D. suzukii* larval development than cultivated fruit despite the higher quantities of secondary metabolites. Additional factors may have influenced female oviposition choice, including plant volatile emission, trace pesticide compounds on the cultivated fruits, or perhaps the higher phytochemical levels in wild fruit provided increased defense against egg or larval parasitoids. Further research is needed to tease apart all interacting factors.

A number of factors contribute to oviposition site selection in herbivores, including previous experience, host condition, competition, and predator avoidance (Carrasco et al., 2015; Futuyma & Peterson, 1985; Jaenike, 1978; Papaj & Prokopy, 1989). The higher oviposition we observed in under‐ripe wild berries may, in part, be related to their relatively shorter ripening times. Wild blackberries are about a quarter the size of the cultivated ‘Ouachita’ variety we sampled, and exhibited a swifter progression from the blush to ripe stage during collection. That *D. suzukii* can and do develop on a wide variety of host plants and even non‐host plants suggests that larval nutritional needs are plastic (Jaramillo et al., 2015; Little et al., 2020; Young et al., 2018). *Drosophila suzukii* avoid laying eggs in overripe fruit, presumably to avoid interspecific competition, so it is reasonable to expect a large spillover effect into under‐ripe berries if a significant determining factor for fitness is competition. The tradeoff to laying eggs into less‐ripe fruit may be small if the appropriate nutrients are gathered as the berry ripens during the same length of time as larvae develop. Wild berries that were blush 1 week were observed to be overripe or gone at the next sampling point 2 weeks later. This strategy might not succeed for egg laying into cultivated blackberries because these fruit take a longer time to fully develop (see Swoboda‐Bhattarai & Burrack, 2015). Although the process of crop domestication reduced or eliminated many inherent plant defenses against insect pests, that *D. suzukii* oviposit more readily into wild fruit suggests that non‐domesticated blackberries are more exploitable than their cultivated counterpart. Except for 1 week, the average berry infestation across all ripeness stages was higher for wild berries than cultivated berries.

Another consequence of fruit domestication is a change in fruit ripening windows. Cultivated crops are selected to produce fruit where the majority will ripen around the same time to reduce harvesting labor cost, leaving less diversity among ripeness stages for female oviposition selection (Heiser, 1988). In cultivated berries, the number of eggs per berry at each ripeness stage over the season were consistently different from each other, suggesting that oviposition was additive at each stage; the number of eggs per berry increased as a function of time and ripeness. Contrastingly, there was no such pattern in the wild berry samples, which is more indicative of a simultaneous rather than sequential infestation. These differences in oviposition may reflect the two environments. Wild blackberries grew in a wooded understory that may offer an increased window of conditions favorable to oviposition, while cultivated berries grew in unfavorable areas fully exposed to the sun. However, *D. suzukii* can also remain in cultivated habitat during the day and exploit microclimates within the crop canopy, thus increasing the oviposition window (Diepenbrock & Burrack, 2017). Regardless, *D. suzukii* exhibit diurnal oviposition behavior, with the majority of eggs being laid in late afternoon and dusk hours (Hamby et al., 2016). Along with our random sampling protocol, any confounding environmental effects were reduced, but not eliminated entirely.

As discussed, there are numerous qualities that differentiate cultivated fruit from their wild‐growing relatives. To begin to tease these factors apart, we directly assessed whether any of the observed difference in natural oviposition among the two cultivation types resulted from host preference rather than population level or environmental factors. In a two‐choice bioassay, females preferred laying eggs in cultivated fruit when exposed to a single berry of each type, which may indicate a size or surface area preference given the physical disparity between the two fruit types. However, when offered an equal weight, corresponding to a single cultivated versus several wild berries, females laid more eggs in wild fruit. Long‐range perception in oviposition site selection relies on several sensory inputs like visual cues; at a distance, clusters of berries may appear as a single, large fruit, which could explain why our lab preference results differed when the set‐up changed (Bernays & Wcislo, 1994). The observed correlation between apparent fruit size and preference agrees with other visual research on *D. suzukii* attraction and its effects on oviposition behavior (Rice et al., 2016). Olfactory cues may have also played a role in the oviposition behavior. For the equal weight assay, 1 to 9 wild berries per single cultivated berry were used so the levels of olfactory semiochemicals released by the wild blackberries may have been stronger, or more preferred than those of the cultivated berry. Additional experiments to isolate effects from visual and olfactory cues will be needed to further assess any preference among cultivated and wild blackberry fruits.

The pattern and timing of infestation we observed in wild‐growing berries from natural habitats in the eastern United States is consistent with research in Hawaii, Europe and Japan that trapped adult *D. suzukii* in montane habitats (Mueller, 2015; Ometto et al., 2013; Santoiemma et al., 2019). In Japan, while most cultivated fruit crops were grown below 600 m, the majority of *D. suzukii* adults were trapped at higher elevations (Ometto et al., 2013). The present study observed *D. suzukii* infestation in a variety of susceptible host plants at several elevations and especially in the most abundant resource, wild blackberry. Once fruit began to ripen, sometime between the green and blush stage, the berries were exploited for oviposition by *D. suzukii* females. Wild berries appeared to develop from blush to ripe in less than 2 weeks (our sampling interval) which aligns with typical immature *D. suzukii* development (Hamby et al., 2016).

Given this information about the presence of established populations of *D. suzukii* in remote, montane regions of the southern Appalachian Mountains, several new research questions arise. Within the wooded landscape, *D. suzukii* may be affecting the local food web by utilizing wild blackberries upstream of other organisms. What effect does this invasive pest have on other blackberry feeders such as birds, bears, or other invertebrates? In terms of agroecosystem impact, do these types of forest populations serve as a potential source for regional migration into crop habitats? Marked *D. suzukii* adults have been caught at distances in excess of four times their flight capacity, suggesting the possibility of movement over long distances (Tait et al., 2018; Wong et al., 2018). The full extent of current and future impacts of *D. suzukii* in croplands and beyond has yet to be fully realized.

## AUTHOR CONTRIBUTIONS

**Johanna E. Elsensohn:** Conceptualization (equal); data curation (lead); formal analysis (equal); funding acquisition (lead); investigation (lead); methodology (equal); project administration (lead); resources (equal); validation (equal); visualization (lead); writing – original draft (lead); writing – review and editing (lead). **Hannah J. Burrack:** Conceptualization (equal); formal analysis (equal); methodology (equal); resources (lead); supervision (lead); validation (equal); writing – review and editing (equal).

## CONFLICT OF INTEREST

The authors declare no conflict of interest.

## Supporting information


Appendix S1

## Data Availability

Data files from this manuscript will be deposited in the Dryad database system available at https://doi.org/10.5061/dryad.6m905qg3x. Data can also be requested through the authors.

## References

[ece39713-bib-0001] Anttonen, M. J. , & Karjalainen, R. O. (2005). Environmental and genetic variation of phenolic compounds in red raspberry. Journal of Food Composition and Analysis, 18, 759–769.

[ece39713-bib-0002] Atallah, J. , Teixeira, L. , Salazar, R. , Zaragoza, G. , & Kopp, A. (2014). The making of a pest: The evolution of a fruit‐penetrating ovipositor in *Drosophila suzukii* and related species. Proceedings of the Royal Society B: Biological Sciences, 281, 20132840. 10.1098/rspb.2013.2840 PMC3953835 24573846

[ece39713-bib-0003] Ballman, E. S. , & Drummond, F. A. (2017). Infestation of wild fruit by *Drosophila suzukii* surrounding Maine wild blueberry fields. Journal of Agricultural and Urban Entomology, 33, 61–70. 10.3954/1523-5475-33.1.61

[ece39713-bib-0004] Barringer, L. , & Ciafré, C. M. (2020). Worldwide feeding host plants of spotted lanternfly, with significant additions from North America. Environmental Entomology, 49, 999–1011. 10.1093/ee/nvaa093 32797186

[ece39713-bib-0005] Bellamy, D. E. , Sisterson, M. S. , & Walse, S. S. (2013). Quantifying host potentials: Indexing postharvest fresh fruits for spotted wing drosophila, *Drosophila suzukii* . PLoS One, 8, e61227. 10.1371/journal.pone.0061227 23593439 PMC3625224

[ece39713-bib-0006] Bellutti, N. , Gallmetzer, A. , Innerebner, G. , Schmidt, S. , Zelger, R. , & Koschier, E. H. (2018). Dietary yeast affects preference and performance in *Drosophila suzukii* . Journal of Pest Science, 91, 651–660. 10.1007/s10340-017-0932-2 29568250 PMC5847167

[ece39713-bib-0007] Bernardi, D. , Andreazza, F. , Botton, M. , Baronio, C. A. , & Nava, D. E. (2017). Susceptibility and interactions of *Drosophila suzukii* and *Zaprionus indianus* (Diptera: Drosophilidae) in damaging strawberry. Neotropical Entomology, 46, 1–7. 10.1007/s13744-016-0423-9 27389188

[ece39713-bib-0008] Bernays, E. A. , & Wcislo, W. T. (1994). Sensory capabilities, information processing, and resource specialization. The Quarterly Review of Biology, 69, 187–204. 10.1086/418539

[ece39713-bib-0009] Calabria, G. , Máca, J. , Bächli, G. , Serra, L. , & Pascual, M. (2012). First records of the potential pest species *Drosophila suzukii* (Diptera: Drosophilidae) in Europe. Journal of Applied Entomology, 136, 139–147. 10.1111/j.1439-0418.2010.01583.x

[ece39713-bib-0010] Carrasco, D. , Larsson, M. C. , & Anderson, P. (2015). Insect host plant selection in complex environments. Current Opinion in Insect Science, 8, 1–7. 10.1016/j.cois.2015.01.014 32846657

[ece39713-bib-0011] Chen, Y. H. , Gols, R. , & Benrey, B. (2015). Crop domestication and its impact on naturally selected trophic interactions. Annual Review of Entomology, 60, 35–58. 10.1146/annurev-ento-010814-020601 25341108

[ece39713-bib-0012] Compton, S. G. (2002). Sailing with the wind: Dispersal by small flying insects. In J. M. Powell , R. E. Kenward , & R. S. Hails (Eds.), Dispersal ecology (pp. 113–133). Blackwell Publishing.

[ece39713-bib-0013] Cosmulescu, S. , Trandafir, I. , & Nour, V. (2017). Phenolic acids and flavonoids profiles of extracts from edible wild fruits and their antioxidant properties. International Journal of Food Properties, 20, 3124–3134. 10.1080/10942912.2016.1274906

[ece39713-bib-0014] de la Vega, G. J. , & Corley, J. C. (2019). *Drosophila suzukii* (Diptera: Drosophilidae) distribution modelling improves our understanding of pest range limits. International Journal of Pest Management, 65, 217–227. 10.1080/09670874.2018.1547460

[ece39713-bib-0015] Deprá, M. , Poppe, J. L. , Schmitz, H. J. , De Toni, D. C. , & Valente, V. L. (2014). The first records of the invasive pest *Drosophila suzukii* in the south American continent. Journal of Pest Science, 87, 379–383. 10.1007/s10340-014-0591-5

[ece39713-bib-0016] Diepenbrock, L. M. , & Burrack, H. J. (2017). Variation of within‐crop microhabitat use by *Drosophila suzukii* (Diptera: Drosophilidae) in blackberry. Journal of Applied Entomology, 141, 1–7. 10.1111/jen.12335

[ece39713-bib-0017] Diepenbrock, L. M. , Swoboda‐Bhattarai, K. A. , & Burrack, H. J. (2016). Ovipositional preference, fidelity, and fitness of *Drosophila suzukii* in a co‐occurring crop and non‐crop host system. Journal of Pest Science, 89, 761–769. 10.1007/s10340-016-0764-5

[ece39713-bib-0018] dos Santos, L. A. , Mendes, M. F. , Krüger, A. P. , Blauth, M. L. , Gottschalk, M. S. , & Garcia, F. R. (2017). Global potential distribution of *Drosophila suzukii* (Diptera, Drosophilidae). PLoS One, 12, e0174318. 10.1371/journal.pone.0174318 28323903 PMC5360346

[ece39713-bib-0019] Elith, J. , & Leathwick, J. R. (2009). Species distribution models: Ecological explanation and prediction across space and time. Annual Review of Ecology, Evolution, and Systematics, 40, 677–697. 10.1146/annurev.ecolsys.110308.120159

[ece39713-bib-0020] Elsensohn, J. E. , & Loeb, G. M. (2018). Non‐crop host sampling yields insights into small‐scale population dynamics of *Drosophila suzukii* (Matsumura). Insects, 9, 5. 10.3390/insects9010005 29301358 PMC5872270

[ece39713-bib-0021] Fitzpatrick, M. C. , Weltzin, J. F. , Sanders, N. J. , & Dunn, R. R. (2007). The biogeography of prediction error: Why does the introduced range of the fire ant over‐predict its native range? Global Ecology and Biogeography, 16, 24–33. 10.1111/j.1466-8238.2006.00258.x

[ece39713-bib-0022] Fraimout, A. , & Monnet, A. C. (2018). Accounting for intraspecific variation to quantify niche dynamics along the invasion routes of *Drosophila suzukii* . Biological Invasions, 20, 2963–2979. 10.1007/s10530-018-1750-z

[ece39713-bib-0023] Futuyma, D. J. , & Peterson, S. C. (1985). Genetic variation in the use of resources by insects. Annual Review of Entomology, 30, 217–238.

[ece39713-bib-0024] Gutierrez, A. P. , Ponti, L. , & Dalton, D. T. (2016). Analysis of the invasiveness of spotted wing drosophila (*Drosophila suzukii*) in North America, Europe, and the Mediterranean Basin. Biological Invasions, 18, 3647–3663. 10.1007/s10530-016-1255-6

[ece39713-bib-0025] Hamby, K. A. , & Becher, P. G. (2016). Current knowledge of interactions between *Drosophila suzukii* and microbes, and their potential utility for pest management. Journal of Pest Science, 89, 621–630. 10.1007/s10340-016-0768-1

[ece39713-bib-0026] Hamby, K. A. , Bellamy, D. E. , Chiu, J. C. , Lee, J. C. , Walton, V. M. , Wiman, N. G. , York, R. M. , & Biondi, A. (2016). Biotic and abiotic factors impacting development, behavior, phenology, and reproductive biology of *Drosophila suzukii* . Journal of Pest Science, 89, 605–619. 10.1007/s10340-016-0756-5

[ece39713-bib-0027] Hardin, J. A. , Kraus, D. A. , & Burrack, H. J. (2015). Diet quality mitigates intraspecific larval competition in *Drosophila suzukii* . Entomologia Experimentalis et Applicata, 156, 59–65. 10.1111/eea.12311

[ece39713-bib-0028] Hassani, I. M. , Behrman, E. L. , Prigent, S. R. , Gidaszewski, N. , Ravaomanarivo, L. R. , Suwalski, A. , Debat, V. , David, J. R. , & Yassin, A. (2020). First occurrence of the pest *Drosophila suzukii* (Diptera: Drosophilidae) in the Comoros archipelago (Western Indian Ocean). African Entomology, 28, 78–83. 10.4001/003.028.0078

[ece39713-bib-0029] Hauser, M. (2011). A historic account of the invasion of *Drosophila suzukii* (Matsumura) (Diptera: Drosophilidae) in the continental United States, with remarks on their identification. Pest Management Science, 67, 1352–1357. 10.1002/ps.2265 21898759

[ece39713-bib-0030] Heiser, C. B. (1988). Aspects of unconscious selection and the evolution of domesticated plants. Euphytica, 37, 77–81.

[ece39713-bib-0031] Hoelscher, C. E. (1967). Wind dispersal of brown soft scale crawlers, *Coccus hesperidum* (Homoptera: Coccidae), and Texas citrus mites, *Eutetranychus banksi* (Acarina: Tetranychidae) from Texas citrus. Annals of the Entomological Society of America, 60, 673–678. 10.1093/aesa/60.3.673

[ece39713-bib-0032] Jaenike, J. (1978). On optimal oviposition behavior in phytophagous insects. Theoretical Population Biology, 14, 350–356. 10.1016/0040-5809(78)90012-6 751265

[ece39713-bib-0033] Jaramillo, S. L. , Mehlferber, E. , & Moore, P. J. (2015). Life‐history trade‐offs under different larval diets in *Drosophila suzukii* (Diptera: Drosophilidae). Physiological Entomology, 40, 2–9. 10.1111/phen.12082

[ece39713-bib-0034] Kamiyama, M. T. , & Guédot, C. (2019). Varietal and developmental susceptibility of tart cherry (Rosales: Rosaceae) to *Drosophila suzukii* (Diptera: Drosophilidae). Journal of Economic Entomology, 112, 1789–1797. 10.1093/jee/toz102 31329912

[ece39713-bib-0035] Kennedy, G. G. , & Storer, N. P. (2000). Life systems of polyphagous arthropod pests in temporally unstable cropping systems. Annual Review of Entomology, 45, 467–493. 10.1146/annurev.ento.45.1.467 10761586

[ece39713-bib-0036] Klick, J. , Yang, W. Q. , Walton, V. M. , Dalton, D. T. , Hagler, J. R. , Dreves, A. J. , Lee, J. C. , & Bruck, D. J. (2016). Distribution and activity of *Drosophila suzukii* in cultivated raspberry and surrounding vegetation. Journal of Applied Entomology, 140, 37–46. 10.1111/jen.12234

[ece39713-bib-0037] Lee, J. C. , Bruck, D. J. , Curry, H. , Edwards, D. , Haviland, D. R. , Van Steenwyk, R. A. , & Yorgey, B. M. (2011). The susceptibility of small fruits and cherries to the spotted‐wing drosophila, *Drosophila suzukii* . Pest Management Science, 67, 1358–1367. 10.1002/ps.2225 21710685

[ece39713-bib-0038] Lee, J. C. , Dreves, A. J. , Cave, A. M. , Kawai, S. , Isaacs, R. , Miller, J. C. , Van Timmeren, S. , & Bruck, D. J. (2015). Infestation of wild and ornamental noncrop fruits by *Drosophila suzukii* (Diptera: Drosophilidae). Annals of the Entomological Society of America, 108, 117–129. 10.1093/aesa/sau014

[ece39713-bib-0039] Little, C. M. , Chapman, T. W. , & Hillier, N. K. (2020). Plasticity is key to success of *Drosophila suzukii* (Diptera: Drosophilidae) invasion. Journal of Insect Science, 20, 5. 10.1093/jisesa/ieaa034 PMC7230767 32417920

[ece39713-bib-0040] Mazzi, D. , & Dorn, S. (2012). Movement of insect pests in agricultural landscapes. Annals of Applied Biology, 160, 97–113. 10.1111/j.1744-7348.2012.00533.x

[ece39713-bib-0041] Mitsui, H. , Beppu, K. , & Kimura, M. T. (2010). Seasonal life cycles and resource uses of flower‐and fruit‐feeding drosophilid flies (Diptera: Drosophilidae) in Central Japan. Entomological Science, 13, 60–67. 10.1111/j.1479-8298.2010.00372.x

[ece39713-bib-0042] Molina, J. M. , Avivar, L. , & Pérez‐Guerrero, S. (2020). Laboratory evaluation of nine highbush blueberry cultivars susceptibility to *Drosophila suzukii* (Matsumura, 1931) in the southwestern Spain. Spanish Journal of Agricultural Research, 18, e10SC03. 10.5424/sjar/2020182-16100

[ece39713-bib-0043] Moser, D. , Drapela, T. , Zaller, J. G. , & Frank, T. (2009). Interacting effects of wind direction and resource distribution on insect pest densities. Basic and Applied Ecology, 10, 208–215. 10.1016/j.baae.2008.03.008

[ece39713-bib-0044] Mueller, M. C. (2015). Islands within islands: The effects of habitat fragmentation, novel community interactions, and climate on Hawaiian drosophila populations. Masters Thesis. University of Hawai'i.

[ece39713-bib-0045] Ometto, L. , Cestaro, A. , Ramasamy, S. , Grassi, A. , Revadi, S. , Siozios, S. , Moretto, M. , Fontana, P. , Varotto, C. , Pisani, D. , & Dekker, T. (2013). Linking genomics and ecology to investigate the complex evolution of an invasive drosophila pest. Genome Biology and Evolution, 5, 745–757. 10.1093/gbe/evt034 23501831 PMC3641628

[ece39713-bib-0046] Ørsted, I. V. , & Ørsted, M. (2019). Species distribution models of the spotted wing drosophila (*Drosophila suzukii*, Diptera: Drosophilidae) in its native and invasive range reveal an ecological niche shift. Journal of Applied Ecology, 56, 423–435. 10.1111/1365-2664.13285

[ece39713-bib-0047] Panel, A. D. , Zeeman, L. , Van der Sluis, B. J. , Van Elk, P. , Pannebakker, B. A. , Wertheim, B. , & Helsen, H. H. (2018). Overwintered *Drosophila suzukii* are the main source for infestations of the first fruit crops of the season. Insects, 9, 145. 10.3390/insects9040145 30360355 PMC6315960

[ece39713-bib-0048] Papaj, D. R. , & Prokopy, R. J. (1989). Ecological and evolutionary aspects of learning in phytophagous insects. Annual Review of Entomology, 34, 315–350.

[ece39713-bib-0049] Parchami‐Araghi, M. , Gilasian, E. , & Keyhanian, A. A. (2015). Spotted wing drosophila, *Drosophila suzukii* (Matsumura) (dip.: Drosophilidae), an invasive fruit pest new to the middle east and Iran. Drosophila Information Service, 98, 59–60.

[ece39713-bib-0050] Pelton, E. , Gratton, C. , Isaacs, R. , Van Timmeren, S. , Blanton, A. , & Guédot, C. (2016). Earlier activity of *Drosophila suzukii* in high woodland landscapes but relative abundance is unaffected. Journal of Pest Science, 89, 725–733. 10.1007/s10340-016-0733-z

[ece39713-bib-0051] Powell, R. A. , & Seaman, D. E. (1990). Production of important black bear foods in the southern Appalachians. Bears: Their Biology and Management, 8, 183–187.

[ece39713-bib-0052] Poyet, M. , Eslin, P. , Héraude, M. , Le Roux, V. , Prévost, G. , Gibert, P. , & Chabrerie, O. (2014). Invasive host for invasive pest: When the Asiatic cherry fly (*Drosophila suzukii*) meets the American black cherry (*Prunus serotina*) in Europe. Agricultural and Forest Entomology, 16, 251–259. 10.1111/afe.12052

[ece39713-bib-0053] Rand, T. A. , Tylianakis, J. M. , & Tscharntke, T. (2006). Spillover edge effects: The dispersal of agriculturally subsidized insect natural enemies into adjacent natural habitats. Ecology Letters, 9, 603–614. 10.1111/j.1461-0248.2006.00911.x 16643305

[ece39713-bib-0054] Rice, K. B. , Short, B. D. , Jones, S. K. , & Leskey, T. C. (2016). Behavioral responses of *Drosophila suzukii* (Diptera: Drosophilidae) to visual stimuli under laboratory, semifield, and field conditions. Environmental Entomology, 45, 1480–1488. 10.1093/ee/nvw123 28028095

[ece39713-bib-0055] Roach, N. S. , Hunter, E. A. , Nibbelink, N. P. , & Barrett, K. (2017). Poor transferability of a distribution model for a widespread coastal marsh bird in the southeastern United States. Ecosphere, 8, e01715. 10.1002/ecs2.1715

[ece39713-bib-0056] Rodriguez‐Saona, C. , Cloonan, K. R. , Sanchez‐Pedraza, F. , Zhou, Y. , Giusti, M. M. , & Benrey, B. (2019). Differential susceptibility of wild and cultivated blueberries to an invasive frugivorous pest. Journal of Chemical Ecology, 45, 286–297. 10.1007/s10886-018-1042-1 30554361

[ece39713-bib-0057] Rossi‐Stacconi, M. V. , Kaur, R. , Mazzoni, V. , Ometto, L. , Grassi, A. , Gottardello, A. , Rota‐Stabelli, O. , & Anfora, G. (2016). Multiple lines of evidence for reproductive winter diapause in the invasive pest *Drosophila suzukii*: Useful clues for control strategies. Journal of Pest Science, 89, 689–700. 10.1007/s10340-016-0753-8

[ece39713-bib-0058] Sakai, A. K. , Allendorf, F. W. , Holt, J. S. , Lodge, D. M. , Molofsky, J. , With, K. A. , Baughman, S. , Cabin, R. J. , Cohen, J. E. , Ellstrand, N. C. , & McCauley, D. E. (2001). The population biology of invasive species. Annual Review of Ecology and Systematics, 32, 305–332. 10.1146/annurev.ecolsys.32.081501.114037

[ece39713-bib-0059] Santoiemma, G. , Fioretto, D. , Corcos, D. , Mori, N. , & Marini, L. (2019). Spatial synchrony in *Drosophila suzukii* population dynamics along elevational gradients. Ecological Entomology, 44, 182–189. 10.1111/een.12688

[ece39713-bib-0060] Santoiemma, G. , Mori, N. , Tonina, L. , & Marini, L. (2018). Semi‐natural habitats boost *Drosophila suzukii* populations and crop damage in sweet cherry. Agriculture, Ecosystems & Environment, 257, 152–158. 10.1016/j.agee.2018.02.013

[ece39713-bib-0061] Sarquis, J. A. , Cristaldi, M. A. , Arzamendia, V. , Bellini, G. , & Giraudo, A. R. (2018). Species distribution models and empirical test: Comparing predictions with well‐understood geographical distribution of *Bothrops alternatus* in Argentina. Ecology and Evolution, 8, 10497–10509. 10.1002/ece3.4517 30464822 PMC6238127

[ece39713-bib-0062] Swoboda‐Bhattarai, K. A. , & Burrack, H. J. (2015). *Drosophila suzukii* infestation in ripe and ripening caneberries. Acta Horticulturae, 1133, 419–430. 10.17660/ActaHortic.2016.1133.65

[ece39713-bib-0063] Tait, G. , Grassi, A. , Pfab, F. , Crava, C. M. , Dalton, D. T. , Magarey, R. , Ometto, L. , Vezzulli, S. , Rossi‐Stacconi, M. V. , Gottardello, A. , & Pugliese, A. (2018). Large‐scale spatial dynamics of *Drosophila suzukii* in Trentino, Italy. Journal of Pest Science, 91, 1213–1224. 10.1007/s10340-018-0985-x

[ece39713-bib-0064] Teulon, D. A. J. , Leskey, T. C. , & Cameron, E. A. (1998). Pear thrips *Taeniothrips inconsequens* (Thysanoptera: Thripidae) life history and population dynamics in sugar maple in Pennsylvania. Bulletin of Entomological Research, 88, 83–92. 10.1017/S0007485300041584

[ece39713-bib-0065] Tonina, L. , Mori, N. , Sancassani, M. , Dall'Ara, P. , & Marini, L. (2018). Spillover of *Drosophila suzukii* between noncrop and crop areas: Implications for pest management. Agricultural and Forest Entomology, 20, 575–581. 10.1111/afe.12290

[ece39713-bib-0066] Van de Velde, F. , Grace, M. H. , Esposito, D. , Pirovani, M. É. , & Lila, M. A. (2016). Quantitative comparison of phytochemical profile, antioxidant, and anti‐inflammatory properties of blackberry fruits adapted to Argentina. Journal of Food Composition and Analysis, 47, 82–91. 10.1016/j.jfca.2016.01.008

[ece39713-bib-0067] Wallingford, A. K. , Rice, K. B. , Leskey, T. C. , & Loeb, G. M. (2018). Overwintering behavior of *Drosophila suzukii*, and potential springtime diets for egg maturation. Environmental Entomology, 47, 1266–1273. 10.1093/ee/nvy115 30124807

[ece39713-bib-0068] Ward, L. K. , & Spalding, D. F. (1993). Phytophagous British insects and mites and their food‐plant families: Total numbers and polyphagy. Biological Journal of the Linnean Society, 49, 257–276.

[ece39713-bib-0069] Weakley, A. S. (2006). Flora of the Carolinas, Virginia, Georgia and surrounding areas. University of North Carolina at Chapel Hill.

[ece39713-bib-0070] Whitehead, S. R. , Turcotte, M. M. , & Poveda, K. (2017). Domestication impacts on plant–herbivore interactions: A meta‐analysis. Philosophical Transactions of the Royal Society B: Biological Sciences, 372, 20160034. 10.1098/rstb.2016.0034 PMC5182430 27920379

[ece39713-bib-0071] Wink, M. (1988). Plant breeding: Importance of plant secondary metabolites for protection against pathogens and herbivores. Theoretical and Applied Genetics, 75, 225–233.

[ece39713-bib-0072] Wong, J. S. , Cave, A. C. , Lightle, D. M. , Mahaffee, W. F. , Naranjo, S. E. , Wiman, N. G. , Woltz, J. M. , & Lee, J. C. (2018). *Drosophila suzukii* flight performance reduced by starvation but not affected by humidity. Journal of Pest Science, 91, 1269–1278. 10.1007/s10340-018-1013-x

[ece39713-bib-0073] Wright, J. W. , Davies, K. F. , Lau, J. A. , McCall, A. C. , & McKay, J. K. (2006). Experimental verification of ecological niche modeling in a heterogeneous environment. Ecology, 87, 2433–2439. 17089652 10.1890/0012-9658(2006)87[2433:evoenm]2.0.co;2

[ece39713-bib-0074] Yilmaz, K. U. , Zengin, Y. , Ercisli, S. , Serce, S. , Gunduz, K. , Sengul, M. , & Asma, B. M. (2009). Some selected physico‐chemical characteristics of wild and cultivated blackberry fruits (*Rubus fruticosus* L.) from Turkey. Romanian Biotechnological Letters, 14, 4152–4163.

[ece39713-bib-0075] Young, Y. , Buckiewicz, N. , & Long, T. A. (2018). Nutritional geometry and fitness consequences in *Drosophila suzukii*, the spotted‐wing drosophila. Ecology and Evolution, 8, 2842–2851. 10.1002/ece3.3849 29531699 PMC5838031

