# Supplementary material for: Plasticity in oviposition and foraging behavior in the invasive pest Drosophila suzukii across natural and agricultural landscapes
Source: Ecol Evol. 2023 Jan 6;13(1):e9713. doi: 10.1002/ece3.9713 (PMC9817201; doi:10.1002/ece3.9713)
Supplement: Supplementary file 1 — Appendix S1 [file ECE3-13-e9713-s001.docx]

**Supplemental Information**

**Table S1.** Collection locations with corresponding habitat and elevation data.

| **Location** | **Habitat** | **Zone** | **Longitude** | **Latitude** | **Elevation (m)** | **Years sampled** |
| --- | --- | --- | --- | --- | --- | --- |
| Southern Nantahala | woods | 17S | 83°01'20.4"W | 35°09'19.5"N | 1230 | 2017 |
| Southern Nantahala | woods | 17S | 83°17'16.5"W | 35°04'33.7"N | 1330 | 2017 |
| Joyce Kilmer | woods | 17S | 83°58'41.81"W | 35°21'50.89"N | 1430 | 2017, 2018 |
| Joyce Kilmer | woods | 17S | 83°59'19.09"W | 35°22'31.46"N | 1630 | 2017, 2018 |
| Joyce Kilmer | roadside | 16S | 84°01'28.0"W | 35°20'16.7"N | 1345 | 2017 |
| Joyce Kilmer | roadside | 16S | 84°00'09.1"W | 35°18'14.3"N | 1630 | 2017 |
| Joyce Kilmer | roadside | 16S | 84°02'07.7"W | 35°20'42.9"N | 1360 | 2017 |
| Joyce Kilmer | roadside | 17S | 84°01'18.8"W | 35°19'03.6"N | 1085 | 2017 |
| Joyce Kilmer | roadside | 17S | 84°02'07.7"W | 35°20'42.9"N | 1500 | 2017 |
| Joyce Kilmer | roadside | 17S | 83°58'57.1"W | 35°19'14.2"N | 1260 | 2017 |
| Joyce Kilmer | roadside | 17S | 83°49'34.1"W | 35°20'07.2"N | 610 | 2017, 2018 |
| Research station | farm | 17S | 81°19'01.5"W | 36°23'59.3"N | 900 | 2017, 2018 |
| Research station | farm | 17S | 82°33'29.7"W | 35°25'05.9"N | 610 | 2017 |
| Cherokee | woods | 17S | 81°49'50.2"W | 36°05'49.6"N | 1630 | 2017 |
| Cherokee | woods | 17S | 81°50'13.6"W | 36°05'23.8"N | 1430 | 2017 |
| Joyce Kilmer | woods | 17S | 83°59'3.62"W | 35°22'50.16"N | 1480 | 2018 |
| Joyce Kilmer | woods | 17S | 83°59'12.08"W | 35°22'41.74"N | 1530 | 2018 |
| Joyce Kilmer | woods | 17S | 83°59'18.13"W | 35°22'33.49"N | 1580 | 2018 |

**Table S2.** Combined season long average eggs per berry in samples of wild-grown blackberries collected in and around JKWA.

| **Ripeness stage** | **Mean ± SE**  **Roadside** | **N** |  | **Mean ± SE**  **Woods** | **N** |
| --- | --- | --- | --- | --- | --- |
| Green | 4.02 ± 0.54 | 51 |  | 4.88 ± 1.0 | 33 |
| Blush | 2.17 ± 0.33 | 46 |  | 5.42 ± 0.26 | 254 |
| Red | 6.67 ± 0.45 | 148 |  | 5.80 ± 0.32 | 196 |
| Purple | 9.16 ± 0.46 | 177 |  | 8.69 ± 0.35 | 245 |
| Ripe | 9.25 ± 0.40 | 178 |  | 11.03 ± 0.39 | 253 |

**Table S3**. Plant species sampled at the ripe stage during study period. Percent infestation is defined as the percentage of berries containing at least one egg.

| **Species** | **Mean eggs per berry ± SE** | **Mean eggs per gram ± SE** | **N** | **Percent infestation** |
| --- | --- | --- | --- | --- |
| *Vaccinium erythrocarpum* | 4.91± 0.32 | 21.16 ± 1.37 | 60 | 100% |
| *Vaccinium pallidum* | 5.9 ± 0.61 | 20.03 ± 2.06 | 20 | 100% |
| *Phytolacca americana* | 12.4 ± 0.8 | 34.51 ± 2.24 | 30 | 100% |
| *Maianthemum racemosum* | 0.33 ± 0.17 | 1.81 ± 0.94 | 21 | 19% |
| *Polygonatum biflorum* | 0 ± 0 | 0 ± 0 | 18 | 0% |

**Figure S1**. Weekly average percentage of fruit infested per sample date in a) cultivated or b) wild-grown blackberries. Symbol markers indicate collection dates for a specific ripeness stage. .
